# DNA Binding with Acetate Bis(1,10-phenanthroline)silver(I) Monohydrate in a Solution and Metallization of Formed Structures

**DOI:** 10.3390/polym9060211

**Published:** 2017-06-08

**Authors:** Nina Kasyanenko, Zhang Qiushi, Vladimir Bakulev, Mikhail Osolodkov, Petr Sokolov, Viktor Demidov

**Affiliations:** 1 Department of Physics, St. Petersburg State University, Universitetskaya Naberezhnaya 3/7, 199037 St. Petersburg, Russia; zqsdxx@163.com (Z.Q.); vbakulev@inbox.ru (V.B.); mio1993@mail.ru (M.O.); p.a.sokolov@spbu.ru (P.S.); 2 Pro-Brite Company, Sofiyskaya ul., 93, 192289 St. Petersburg, Russia; vndemidov@mail.ru

**Keywords:** DNA, bis(1,10-phenanthroline)silver(I) monohydrate, DNA fibrils with silver, silver reduction on DNA

## Abstract

The study of DNA interaction with the acetate bis(1,10-phenanthroline)silver(I) monohydrate in a solution is of interest both for understanding the mechanism of biological activity of silver compound and for forming ordered structures (DNA fibrils) that can be used to solve various problems in the field of nanotechnology. The analysis of changing the DNA conformation (secondary structure, persistent length and volume effects) during the interaction by the methods of UV spectroscopy with the analysis of DNA melting, circular dichroism, viscosity, flow birefringence, AFM (atomic force microscopy) and SEM (scanning electron microscopy) was performed. The formation of two types of complexes was observed. At lower concentration of compound in DNA solution, silver atoms form the coordination bonds with a macromolecule, while the released phenanthroline ligands intercalate between DNA bases. When the concentration of the compound increases, the phenanthroline ligands form an ordered “layer” around the helix. The excess of silver compounds in the DNA solution (with more than five silver atoms per base pair), DNA precipitation is observed with the formation of long fibrils. It was shown that the binding of silver to DNA during the formation of complexes provides further metallization of the resulting structures with the aid of reducing agents; phenanthroline ligands influence the result of such metallization.

## 1. Introduction

The usage of a DNA molecule to create various structures for nanoelectronics has become a fairly common technique. In relation with this, there is often a need for DNA metallization. Most often, natural DNA or synthetic polynucleotide chains are used for binding of modified metal nanoparticles, or, for example, as a template on which the reduction of pre-bound metal ions occurs [1,2,3,4].

Often, in order to solve specific problems, it is necessary to reach a uniform coverage of macromolecules or fibrils with a metal layer, with thickness satisfactory to obtain and use plasmon resonance for various applications. This study examines one of the ways to solve this problem by metallizing DNA through the reduction of silver after the formation of DNA complexes with silver compounds containing phenanthroline ligands. 

The study of DNA interaction with the compound used in the research is also of interest because of the biological activity of silver complexes with phenanthroline [5,6]. Indeed, due to the biological activity of silver, its complexes with different heterocyclic ligands can be used in treatment of serious diseases [7,8]. In addition, silver is regarded as a powerful tool that increases the immunity and actively inhibits the growth of pathogenic bacteria and viruses [9]. The greatest effect on the pathogenic bacteria is achieved when silver is used in a colloidal state [10]. Highly dispersed colloidal particles slowly release silver ions into the solution that prevents the rapid inactivation of silver due to the interaction with the medium components. Combination of silver nanoparticles with drugs enhances the therapeutic effect. In some cases, silver nanoparticles can be used for the improvement of anticancer treatment [11,12,13,14]. Silver complexes with ligands can prolong the action of silver and protect its rapid inactivation [15]. It should be noted that silver is toxic in large quantities. Metallic silver is less toxic than silver in the ionic form. The incorporation of silver compounds and silver nanoparticles into nanocontainers is a convenient way to protect silver and to get effective treatment with minimal side effects [16]. The self-assembly of silver compounds with different ligands into polymolecular assemblies can be observed. Weak supramolecular forces such as hydrogen bonds and π–π stacking stabilize these structures. Silver compounds with appropriate ligands are able to generate coordination polymers, which contain metal cation centers linked by organic ligands. Coordination polymers have wide applications, including medical treatments [17,18,19,20,21]. The polyatomic structures were observed for silver compounds with phenanthroline and its derivatives [22,23,24,25]. The biological activity of the metal coordination complexes with phenanthroline, including anticancer properties, is well known [26,27,28].

The interest to fabrication and control the properties of silver nanoparticles, silver nanoclusters and thin silver films is determined by their applications in nanotechnology in creating structures and devices for different purposes. Indeed, the localized surface plasmon resonance (LSPR) of silver nanoparticles can be used in biosensors, controlling devices and in other systems [29,30]. Luminescence silver nanoclusters consisting of less than 20 atoms are of significant interest for bioimaging [31,32]. Sodium borohydride, ascorbic acid, monosaccharides and certain polymers [33,34] can reduce silver ions. The conjugation of silver nanoparticles with synthetic and biological polymers is of active use for the development of molecular medicine, nanoelectronics and nanooptics [35,36]. The DNA molecule is a good template for the formation of silver nanoclusters and nanoparticles [37,38]. 

The investigation of the interaction of silver and its compounds with biopolymers in vitro is a key stage in the understanding of their biological activities. It was mentioned that complexes of metals with 1,10-phenanthrolines interact with DNA as intercalators [39,40]. They can also be connected to DNA via the coordination bonds [41]. It was shown that 1,10-phenanthrolines in complexes with redox active metals, e.g., Cu(II)/Cu(I), Co(II)/Co(III), Fe(II)/Fe(III), can participate in oxidative damage of DNA [42]. Metal complexes with phenanthroline have been tested as potential anticancer drugs [43,44], and 1,10-phenanthroline complexes of Cu(I) and Cu(II) inhibit human immunodeficiency viruses [45]. 

In our study, we use complex of Ag (I) with 1,10-phenanthroline. DNA interaction with similar silver compounds was studied previously [46,47,48]. Note that several Ag (I) complexes have shown good activity against different types of tumors [49,50,51,52]. In this connection, it is of great interest to study the possibility of Ag–Phen interaction with DNA, since DNA is the main target for majority of antitumor drugs in the cell. Such studies are very convenient to carry out using model systems—water–salt solutions of DNA. 

In our research, we use acetate [Ag(Phen)_2_]OAc·H_2_O (hereinafter referred to as Ag–Phen). It should be noted that the acetate bis-compound of Ag (I) with 1,10-phenanthroline is much more soluble in water than its nitrates. Therefore, aqueous solutions of DNA are a good model system for studying the molecular mechanism of the biological effect of the compound. In addition, the silver compounds interacting with DNA are always of interest because of the possible metallization of the macromolecule. DNA metallization is widely used in new biotechnological developments [35,36,37,38]. The aim of the study is to compare the interaction of high molecular DNA with silver ions, 1,10-phenantroline and Ag–Phen in a solution, as well as metallization of DNA in a solution by addition of reducing agent after the formation of DNA complexes with silver compounds.

It is well known that the Ag^+^ ions can be quite easily reduced to metallic silver in water by various reducing agents. The reduction of silver ions after their binding to DNA gives the possibility of DNA metallization. In this case, we can observe silver nanoparticles attached to DNA chains [37,38]. When using complexes of the Ag–Phen with DNA in aqueous solution, we expect that phenanthroline ligands will provide a more even fixation of silver on DNA, so that instead of discrete nanoparticles, a thin layer of silver can be obtained on macromolecular fibrils. We analyze the features of silver reduction in complexes with DNA after the binding of Ag–Phen and make a comparison with the result of DNA metallization using AgNO_3_. 

Thus, the study had two goals: to consider the molecular model of DNA binding with Ag–Phen exhibiting biological activity (preliminary data showed its potential cytostatic activity) and to carry out reduction of silver in the DNA–Ag–Phen complexes with further analysis of the formed structures and their comparison with the results of DNA metallization in complexes with Ag^+^.

## 2. Materials and Methods

**Figure 1:**
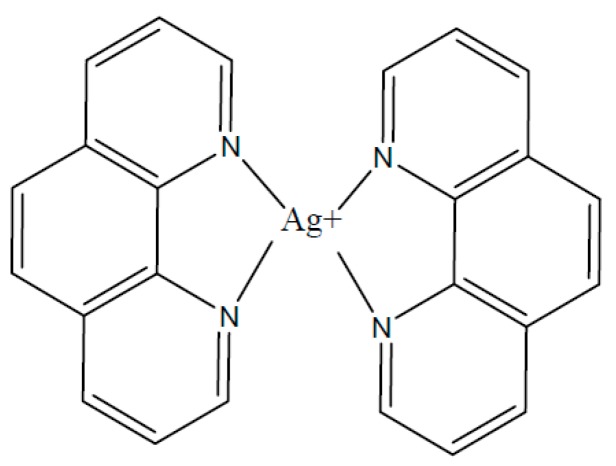
The structure of [Ag(Phen)_2_]^+^ cation. The nearest neighbors of the silver ion Ag^+^, N(1), N(2), N(3), and N(4), form distorted tetrahedron, in which the ligand planes are rotated relative to each other, according to experimental data [47,48].

Calf thymus DNA (Sigma-Aldrich, Darmstadt, Germany) was dissolved in distilled water. After 5 days of storage at 4 °C, the solution of NaNO_3_ was mixed with DNA solution in order to achieve the desired concentration (typically 0.005 M). Stock DNA solutions in 0.005 M NaNO_3_ were centrifuged or filtered before usage. The molecular mass of DNA (9 × 10^6^) was determined from the value of DNA intrinsic viscosity [η] in 0.15 M NaCl. For comparison the synthesis of free silver nanoparticles was carried out with AgNO_3_ and NaBH_4_. The coordination compound of silver (I) with 1,10-phenanthroline, bis(1,10-phenanthroline)silver(I) acetate monohydrate, [Ag(Phen)_2_]OAc·H_2_O, conventionally referred to as Ag–Phen (Figure 1), was prepared according to the procedure described in [47].

### 2.1. Synthesis of Bis(1,10-phenanthroline)silver(I) Acetate Monohydrate, [Ag(phen)_2_]OAc·H_2_O

A solution of Na_2_CO_3_ (14.72 mmol) was added dropwise under vigorous stirring to a solution of AgNO_3_ (29.44 mmol) at room temperature. The yellow precipitate Ag_2_CO_3_ was washed with water, and acetic acid AcOH was added for preparing of 50% aqueous solution. The mixture was stirred until there was no more evolution of carbon dioxide CO_2_, and then the resulting colorless mass of silver (I) acetate AgOAc was evaporated at 100 °C twice with the addition of water. Finally, silver (I) acetate was dissolved in water with equivalent amount of 1,10-phenanthroline hydrate (58.88 mmol). The mixture was heated at 80–90 °C and stirred for 2 h to form a bright yellow solution. The complex [Ag(Phen)_2_]OAc was evaporated. It was found: C 57.50%, H 3.62%, N 10.44%. The calculation from structural formula [Ag(Phen)_2_]OAc·H_2_O, [Ag(C_12_H_8_N_2_)_2_]CH_3_COO·H_2_O gives: C 57.27%, H 3.88%, N 10.27%.

Sodium borohydride was dissolved in distilled water immediately prior to its use. In aqueous solutions of Ag–Phen complexes, the balance among [Ag(Phen)_2_]^+^, [Ag(Phen)(H_2_O)_2_]^+^ and [Ag(Phen)(OAc)] has been gradually established.

A high molecular calf thymus DNA (Sigma) was used. The molecular mass of DNA, *M* = 10^7^ was determined from the value of the DNA intrinsic viscosity [η] (in dL/g) in 0.15 M NaCl with the formula [53]:

$$\left[\eta \right]=6.9\times 10^{-4}\times M^{0.7}$$

DNA was dissolved in distilled water, and after 5 days of storage at 4 °C a certain amount of salt solution was added to achieve 0.005 M NaCl. Then the DNA solution was centrifuged and filtered. The DNA concentration in a stock solution was determined after the DNA hydrolysis in 6% HClO_4_ at 100 °C for 15 min from the difference in the absorbance Δ*D* at two wavelengths 270 and 290 nm: *C*(DNA) = (*D*_270_ − *D*_290_) × 5 × 10^−3^, % [54]. This approach along with the pre-denaturation of DNA allows us to control the value of molar extinction coefficient in complexes with the absorption of native DNA at 260 nm: *E*_260_(P) = 31.1 × *D*_260_/*C*(DNA), %. 

### 2.2. Viscosimetry

The relative solution viscosity $\eta =\frac{\eta }{\eta_{0}}$ (where η and η_0_ are the viscosities of the solution and the solvent, respectively) was measured at different velocity gradients *g* in the range of 0.5–2 s^−1^. The use of η*_r_* value at *g*→0 gives the reduced viscosity of the DNA solution $\eta_{red}=\frac{\eta_{r}-1}{c}$ whose dependence on DNA concentration *c* allows us to determine the DNA intrinsic viscosity [η]:

$$\left[\eta \right]=lim_{c\to 0}\frac{\left(\eta_{r}-1\right)}{c}$$

For DNA with *M* > 2 × 10^6^, the model of swelling statistical coil can be used. The Kuhn’s model of polymer chain consisting of freely jointed segments is suitable for DNA in this case. The length of the segment *A* indicates the chain rigidity and is related to the DNA persistent length *p* by the equation *A* = 2*p*. The value [η] is related to DNA parameters by the Flory’s formula:

(1) $$\left[\eta \right]=\Phi \;\frac{{\langle h^{2}\rangle }^{3/2}}{M}=\Phi \;\frac{{\langle L2p\rangle }^{3/2}}{M}\alpha^{3}$$

where Ф is the Flory parameter, *M* is the molecular mass, *L* is the hydrodynamic length of the DNA molecule, α is the coefficient of linear swelling describing the volume effect including the polyelectrolyte swelling. The parameter <*h*^2^>^1/2^ (the mean square distance between the ends of the polymer chain) defines the linear size of the molecular coil, in a solution <*h*^2^>^1/2^ = α (*LA*)^1/2^.

In our research we use the viscosimetric titration. The dependence of the reduced viscosity of a DNA solution on the concentration of compounds at constant concentration of DNA was studied. The studies can reveal the influence of binding on the volume of the macromolecule, which is determined by the bending chain rigidity (DNA persistent length *p*) and by the polyelectrolyte swelling α at constant *M* and *L*. The decrease in the reduced viscosity indicates the decrease in the volume of the DNA molecular coil. 

### 2.3. Flow Birefringence

The birefringence values Δ*n* for solutions with different DNA concentrations *C* were measured in the field of the velocity gradient *g*, which provides the orientation of ellipsoidal molecular coils. The value (Δ*n*/*g*)/*c*η_0_ for *g→*0 and *c→*0 was used to calculate the dynamooptic constant [*n*] which can determine the optical anisotropy of the statistical segment (α_1_ − α_2_) i.e., the difference between the polarizabilities of the segment along and across the axis of the DNA helix. For the Kuhn’s model of freely jointed chain, we have:

(2) $$\frac{\left[n\right]}{\left[\eta \right]}=\frac{4\pi }{45kTn_{s}}\frac{{\left(n_{s}^{2}-1\right)}^{2}}{n_{s}}\left(\alpha_{1}-\alpha_{2}\right)$$

where [η] is the DNA intrinsic viscosity. For DNA the following equation is valid:

(3) $$\frac{{\left(\Delta n/g\right)}_{g\to 0}}{\left(\eta_{r}-1\right)\eta_{0}}=\frac{\left[n\right]}{\left[\eta \right]}=\frac{4\pi }{45kTn_{s}}\frac{{\left(n_{s}^{2}-1\right)}^{2}}{n_{s}}\left(\alpha_{1}-\alpha_{2}\right)$$

Thus, after determining the value $\frac{{\left(\Delta n/g\right)}_{g\to 0}}{\left(\eta_{r}-1\right)\eta_{0}}$ we can estimate the optical anisotropy of the statistical segment:

(4) $$\left(\alpha_{1}-\alpha_{2}\right)=S\Delta \beta =\frac{A}{l}\Delta \beta$$

where *S* is the number of base pairs in the statistical segment with the length *A*, *l* is the base pair length along the DNA axis, *A* = 2*p* (*p* is the persistent length of DNA), and Δβ is the optical anisotropy of a base pair along and normal to the axis of the DNA helix. All hydrodynamic measurements were performed at temperature 21 °C.

### 2.4. Spectral Methods

UV absorption spectra of DNA and its complexes were recorded using a SF-56 spectrophotometer (LOMO, St.-Petersburg, Russia). DNA denaturation, also called DNA melting, was studied with Specord 200 Plus equipped with the Peltier temperature controlled cell holder. Melting curves of DNA in complexes were registered at 260 nm. The optical path length in quartz cuvettes was 1 cm. The melting temperature of DNA that refers to the temperature at which 50% of DNA has denatured from double-stranded to single-stranded DNA (the average temperature of the helix–coil transition) was derived from the peak maximum of first deviation of the melting curves.

### 2.5. Circular Dichroism

Spectra were recorded with Mark IV Autodichrograph (Jobin Ivon, France). ∆ε = ∆*D*/*cd* (Δ*D* is the difference between the optical densities for left and right circular polarized light, *c* is the DNA molar concentration, *d* is the optical pathway. 

The luminescence was measured with a Hitachi-850 fluorescence spectrometer (Tokyo, Japan) using a 1 cm-thick quartz cuvette after the solutions were held for 1 h at the ambient temperature. Luminescence excitation and the emission spectra were corrected for the spectral sensitivity of the instrument. 

### 2.6. Atomic Force and Scanning Electron Microscopy

The AFM images of DNA, its complexes with Ag–Phen and metallized structures were obtained on the surface of silicon or mica with AFM Bruker Multimode 8 equipped with Nanoscope V controller (Massachusetts, MA, USA) in ScanAssist in air mode. The samples were placed on silicon or freshly cleaved mica substrates. The SEM images were obtained by SEM Carl Zeiss Merlin and SEM Carl Zeiss Auriga (Oberkochen, Germany) using Inlens, SE2 and ESB detectors. The samples were placed on silicon substrates, previously cleaned using ultrasonic cleaning in acetone, ethanol and water and plasma cleaning in gases mixture containing 95% argon and 5% oxygen by Fischione NanoClean Model 1070 for 10 min.

## 3. Results and Discussion

When considering the interaction of Ag–Phen compound with DNA, it is important to understand how the binding affects the secondary and tertiary structure of the macromolecule. Changes in the secondary structure are usually reflected in the absorption and CD spectra of DNA, while the state of the tertiary structure is conveniently analyzed using hydrodynamic methods. Scanning microscopy can also provide useful information on the DNA conformational changes during the interaction with the compound being studied. 

**Figure 2:**
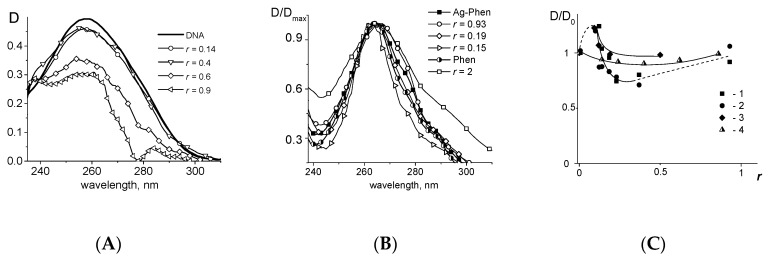
Calculated absorption spectra of DNA (**A**); and normalized spectra of Ag–Phen (**B**) in complexes at different *r* (*r* values are given near lines). Spectra of free Ag–Phen and Phen are also presented. Dependences of relative change in Ag–Phen (1, 2) and Phen (3) absorption at 265 nm (1, 3) and 277 nm (2) in complexes with DNA and relative change in DNA absorption at 260 nm in complexes with Ag^+^ (4) on *r* value are shown in (**C**). *D*_0_ is an adsorption at *r* = 0.

The absorption spectra of Ag–Phen compound and its ligand–phenanthroline (Phen) overlap the DNA absorption band. In this connection, we consider calculated DNA absorption spectra in complexes, assuming unchangeable spectral properties of Ag–Phen compounds when bonded to DNA. The hypochromic effect without shift of the DNA absorption band in complexes with Ag–Phen was observed at *r* ≤ 0.4, where r value indicates the number of silver atoms per one DNA base pair (Figure 2A). At *r* > 0.4 one can see the transformation of the “shoulder” in the long-wave region of the spectrum into the additional peak. A huge hypochromic effect and significant changes in the shape of the calculated DNA absorption band cannot be related in this case only to DNA absorption: the experimental results indicate the transformation of Ag–Phen spectrum in complexes with DNA as well. For example, the binding of Ag–Phen to DNA at *r* > 0.4 is characterized by the manifestation of an alternative type of complexes, and can be accompanied by a stacking of chromophores (Phen ligands), that reduces the absorption of complexes. In addition, the shape of Ag–Phen spectrum is changed in this case. Calculated and normalized spectra of Ag–Phen in complexes with DNA (Figure 2B) show two trends: the narrowing of the peak at *r* ≤ 0.4, and a small bathochromic shift of maximum with the broadening of the band at *r* > 0.4. For small *r* value (excess of DNA), the spectrum is close to that observed for free 1,10-phenanthroline (Phen), although there exist some differences. It could indicate that under these conditions, binding of Ag–Phen to DNA releases one phenanthroline ligand that can interact with DNA regardless of the bound silver atom. At higher r, the alternative state of phenanthroline ligands in complexes is manifested (exactly Phen absorption is mainly observed for Ag–Phen). The alternative DNA–Ag–Phen complexes with the participation of Phen ligands remaining in the silver coordination sphere appear. The dependence of relative change of band amplitude for Ag–Phen and free 1,10-phenantroline absorption in complexes with DNA (Figure 2C) suggests that the binding at low *r* can induce the destruction of phenanthroline stacks, which, apparently, exist in solutions without DNA. For Ag–Phen–DNA complexes the change in DNA absorption is also observed due to silver binding with base pairs. At higher *r* (*r* ≥ 0.4) an increase in the absorption of Ag–Phen differs from the gradual growth of Phen absorption. Thus, experimental results indicate 2 types of Ag–Phen–DNA complexes. It should be noted that the dependence of relative change in Ag–Phen absorption at 277 nm can reflect the DNA interaction with silver. Indeed, DNA spectrum at *C*(Ag–Phen) = 0 was subtracted from the total spectra of complexes for the analysis of Ag–Phen absorption, but really DNA absorption depends on r value. The DNA absorption spectra in complexes with Ag^+^ (while using AgNO_3_) show bathochromic shift with hypochromic effect at 260 nm and small hyperchromic effect at 277 nm (see Supplementary Materials, Figure S1). That indicates binding of silver ions to N7 Guanine [55,56,57]. Silver ion linkage with DNA bases actually affects the electron density of heterocycles, followed by some instability in DNA secondary structure. If the number of such binding sites is large, hydrogen bonds and base stacking are disturbed, and the hyperchromic effect in the DNA absorption spectra appears. The binding of Ag–Phen to DNA in concentrations used stabilizes the double helix. 

When binding Ag–Phen to DNA, silver coordinates to N7 guanine, one Phen ligand emerges into the solution, and another related to silver Phen lays on the periphery of the helix. We can suppose the formation of phenanthroline stacks on the surface of DNA helix at high *r*, and we can not also exclude the intercalation of phenanthroline that can accompany such binding. The intercalation of Phen can be observed because the binding of Ag–Phen to DNA really releases at least one Phen ligand. The excess of binding sites on DNA (at high *C*(DNA) and low *r* value) facilitates such binding as well as makes not very conducive to the formation phenanthroline stacks outside helix. When the number of binding sites on DNA becomes lower (decrease of DNA concentration), the binding turns Phen into the dimeric form.

**Figure 3:**
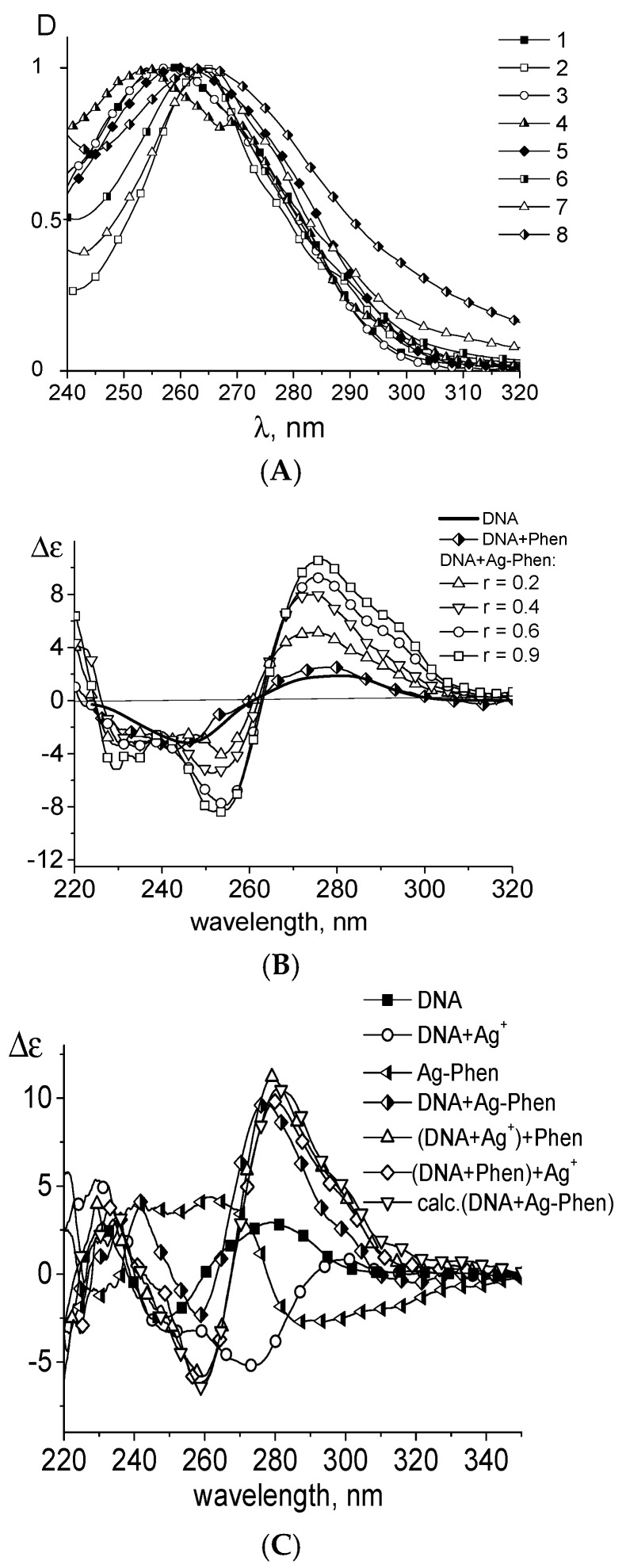
Normalized absorption spectra (**A**) of: DNA (1); Phen (2); Ag^+^ (5); Ag–Phen (7); Phen in AgNO_3_ solution (6); DNA in complexes with Phen (3); DNA in complexes with Ag–Phen (4); and DNA in a solution with Phen and AgNO_3_ (8). CD spectra (**B**,**C**) of DNA in 5 mM NaNO_3_ in complexes with Ag–Phen at different: *r* (**B**); and CD spectra (**C**) of DNA in complexes with Ag^+^, Ag–Phen, and Phen. The results of an addition of AgNO_3_ to DNA+Phen solution [(DNA+Phen)+Ag^+^] and an addition of Phen to DNA+Ag^+^ solution [(DNA+Ag^+^)+Phen] and the result of subtracting of free Ag–Phen spectrum from the spectrum of DNA in complexes with Ag–Phen [calc.(DNA+Ag–Phen)] are shown. *C*(DNA) = 0.0025%, the solvent—5 mM NaNO_3_.

DNA complexation with Ag–Phen differs both from DNA–Ag^+^ and DNA–Phen binding (see Figure 3A). Note that for comparison we use Phen concentration twice as large than Ag–Phen concentration. We can conclude that the binding of Ag–Phen to DNA does not cause a full destruction of silver coordination compound. Thus, at least one Phen ligand remains in the coordination sphere of silver in DNA–Ag–Phen complexes. When AgNO_3_ (as a source of silver ions) and 1,10-phenanthroline were simultaneously added into the DNA solution, the DNA spectrum reflects the result of Ag^+^ binding to DNA. 

Circular dichroism (CD) spectra of DNA in complexes with Ag^+^, Ag–Phen, and Phen also show the difference in their binding (Figure 3B,C). One can see that when the concentration of silver nitrate in the DNA solution increases, the CD spectrum of DNA transforms significantly (see Figure S1 in Supplementary Materials and Figure 3C). CD spectra of DNA in complexes with Phen are close to that for a free DNA. The significant growth in amplitude of positive and negative bands of CD spectrum is observed for DNA at high concentration of Ag–Phen (Figure 3B). We would like to note the appearance of the own CD of Ag–Phen compound when its concentration in the solution is higher than 1.4 × 10^−5^ M. The contribution of the own CD spectrum of Ag–Phen into CD spectrum of the complex is observed in the negative band of CD spectrum (Figure 3B,C). We can assume that the positive band of CD spectrum as a peak at (242 ± 2) nm for free Ag–Phen is presented in DNA solution at high concentration of Ag–Phen. Indeed, after the subtracting of spectrum 3 from spectrum 4, the calculated CD spectrum has approximately equal positive and negative bands. This could be the result of a certain orientation of phenanthroline ligands during Ag–Phen binding to DNA.

Somewhat surprising result was observed for DNA in a solution containing Phen and AgNO_3_. The alternative order of adding of these compounds into DNA solution causes similar CD spectra, which differ only very slightly from the spectrum of DNA–Ag–Phen complex. Indeed, the only difference is the absence of inclusion of CD of free Ag–Phen compounds. Spectra coincide with the calculated spectrum of DNA–Ag–Phen complexes in the absence of free Ag–Phen compounds. We can state that the presence of Ag^+^ and Phen in DNA solution ensures the formation of ternary complexes of DNA–Ag–Phen similar to those observed for DNA interaction with Ag–Phen compound. However, complete coincidence of the formed complexes is not observed, as evidenced by the absorption spectra of the same systems. CD spectra only indicate that the phenanthroline ligands easily interact with silver ions bound to DNA and their orientation is ordered with respect to the plane of nitrogenous bases of DNA. Phen heterocycles form a “coat” around the double helix. Such an orientation may explain the exciton type of CD spectra, and the character of phenanthroline binding to silver does not influence on result. In other words, it is not necessary for phenanthroline to enter the coordination sphere of silver, as in the case of Ag–Phen compound. Note that Phen leaves the coordination sphere of silver when Ag–Phen forms a complex with DNA. This property of phenanthroline can play an important role in the metallization of DNA after the formation of Ag–Phen–DNA complexes. Indeed, after the removal of phenanthroline, silver atom in the complex ion is able to form coordination bonds with the atomic groups of nitrogenous bases. This ensures the reduction of silver on DNA strands. Phenanthroline can stabilize the structure.

**Figure 4:**
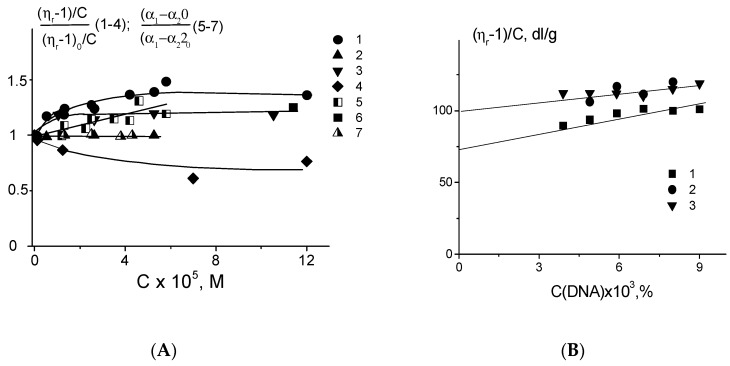
Relative change in reduced viscosity of DNA solutions (1–4) and optical anisotropy of DNA statistical segment (5, 6, 7) with increasing concentration of Ag–Phen (1, 5), AgNO_3_ (2, 7), Phen (3), and Ag–Phen with the addition of NaBH_4_ (4, 6) in 0.005 M of NaNO_3_. (**A**); dependence of reduced viscosity of DNA solutions in 0.005 M NaCl on *C*(DNA) (3) and the result of different dilution of the stock solution containing DNA complexes with Ag–Phen prepared at *C*(Ag–Phen) = 2 × 10^−5^ M and *C*(DNA) = 0.009%, in 0.005 M NaNO_3_, using 0.005 M NaNO_3_ (1) and *C*(Ag–Phen) = 2 × 10^−5^ M in 0.005 M NaNO_3_ (2) as a diluent (**B**).

The reduced viscosity of DNA solutions increases with the rise of Ag–Phen concentration similar to that observed for 1,10-phenanthroline binding to DNA (Figure 4A). It is known that the viscosity of DNA solution may increase due to an intercalation of heterocyclic compounds. We can assume such binding for phenanthroline. Indeed, at least one phenanthroline ligand released from Ag–Phen can intercalate between the bases after silver binding to DNA. The saturation of such binding leads to the cessation of viscosity growth. Figure 4A shows that viscometric results agree with the data of spectral studies. 

The hydrodynamic length of DNA molecule *L* increases due to the intercalation of Phen. According to the neighbor-exclusion principle (a well-known rule for intercalative binding of small planar molecules to DNA) every second (next-neighbor) intercalation site on DNA double helix remains unoccupied. It follows that *L* value can grow up only to a certain limit. This determines an increase in the volume of DNA molecular coil, and in the viscosity during the intercalation. Note that the growth of viscosity is too large for intercalation (see Equation (1)). However, it is necessary to take into account the possible change in chain rigidity (grows of DNA persistent length).

Indeed, the observed increase (Figure 4A, curve 5) in optical anisotropy of DNA statistical segment (α_1_ − α_2_) can be determined by the DNA rigidity (persistent length) and base pair optical anisotropy Δβ (Equation (4)). It should be emphasized, that when studying the optical anisotropy of DNA, we can ignore the shape effect, which is two orders of magnitude smaller than the huge intrinsic optical anisotropy of the macromolecule. Thus, instead of the Kuhn equation (Equation (2)), we use Equation (3). The measured (α_1_ − α_2_) increase can confirm the growth of DNA persistent length during binding and explain the greater increase in viscosity compared to that observed for DNA complexes with phenanthroline. On the other hand, the relative growth of (α_1_ − α_2_) (really this value is negative for DNA due to the negative Δβ value) may indicate also the inclusion of Phen ligands to the measured DNA optical anisotropy. DNA in B-form has a maximal absolute magnitude of the average base pair optical anisotropy ∆β due to the normal orientation of their planes with respect to the axis of the DNA helix. Thus, the observed changes in (α_1_ − α_2_) can be associated with the increase in DNA persistent length or by the contribution of the anisotropy of the Phen ligands to measured ∆β value. In the second case we can assume a predominantly parallel orientation of the phenanthroline planes relative to the plane of bases. As mentioned above, the regular location of Phen heterocycles on DNA helix can explain the observed changes in CD spectra upon DNA complexation with Ag–Phen. Nevertheless, the rigid orientation of phenanthroline relative to base pairs is unlikely. In this connection, it remains to be assumed that the binding of Ag–Phen to DNA causes an increase in the rigidity of the macromolecule (DNA persistent length) about 25%. 

The coordination of silver ions to N7 guanine does not change the DNA optical anisotropy and solution viscosity within used AgNO_3_ concentrations (see Figure 4A, curves 2 and 7). It was also shown that the binding of 1,10-phenanthroline to DNA does not affect the measured optical anisotropy of DNA segment (data is not shown). Thus, at low *C*(Ag–Phen), the binding of silver to DNA releases Phen ligands. Phenanthrolines intercalate independently, and at high *C*(Ag–Phen) after the filling of all possible sites for the intercalation, the additional Ag–Phen binding provides an arrangement of Phen ligands outside bases. Intercalation of Phen does not induce increase of (α_1_ − α_2_). 

Experimental data show that DNA interaction with Phen ligands is observed in all systems. The coordination of silver to DNA is also happens. As we know, the energy of coordination bonds of some metals with DNA is close to the energy of covalent bonds. A weaker linkage of compounds with DNA in a solution depends greatly on the ratio of free and bound fractions of small compounds in DNA solution. On the contrary, the coordination linkage cannot be destroyed by changing of this ratio during the experiment (the formation of coordination bonds requires about 3 h at 21 °C). The next experiment allows us to determine the nature of Ag–Phen–DNA binding (Figure 4B). Measurements were taken in one day after preparing a stock DNA solution with *C*(DNA) = 0.009% (1.36 × 10^−4^ M (bp)) and *C*(Ag–Phen) = 2 × 10^−5^ M (*r* = 0.15) in 0.005 M NaNO_3_. The stock solution was diluted in two different ways: with 0.005 M NaNO_3_ that provides the constant ratio of the concentrations: *C*(Ag–Phen)/*C*(DNA) = const; and with *C*(Ag–Phen) in 0.005 M NaNO_3_ that gives *C*(Ag–Phen) = const. In both cases the relation between DNA-bound fractions of Ag–Phen and free compounds in DNA solution was disturbed. Nevertheless, one can see similar dependences of reduced viscosity on *C*(DNA) (Figure 4B). Thus, two different ways of dilution produce one type of dependence. Its extrapolation to zero DNA concentration (the procedure required to determine the DNA intrinsic viscosity) gives [η] = (100 ± 5) dL/g for DNA in complexes with Ag–Phen and [η] = (86 ± 4) dL/g for free DNA in 0.005 M NaNO_3_. This result is in good agreement with data presented in Figure 4A. Thus, Figure 4B proves the coordination of silver to DNA. Indeed, destruction or an additional formation of coordination bonds does not have time to happen during the experiment.

The melting of DNA in complexes with Ag–Phen shows that the binding causes a certain stabilization of DNA secondary structure that confirms the above assumption. The melting temperature (see Figure S2 in Supplementary Materials) tends to increase with the rise of *C*(Ag–Phen) (see Table 1). Similar data were obtained for DNA complexes with Ag^+^ at the same silver concentrations. Phenanthroline does not show such influence on DNA stability.

**Figure 5:**
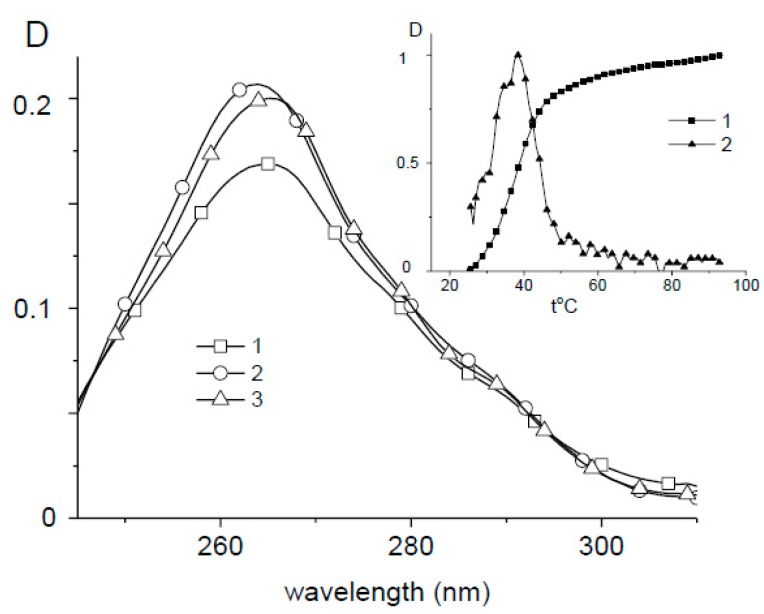
Absorption spectra of Ag–Phen solution without DNA at 25 °C (1), 95 °C (2) and after cooling of solution 2 to 25 °C (3). Insert: The melting curve and its 1st derivative for Ag–Phen in 0.005 M, *C*(Ag–Phen) = 6 × 10^−6^ M.

It is interesting to note that for Ag–Phen solution without DNA at *C*(Ag–Phen) = 6 × 10^−6^ M in 0.005 M NaNO_3_ we have seen hyperchromism at temperature about 38–40 °C that reflects the initial association of Phen ligands with stacking interactions (Figure 5). After cooling, we do not observe total reconstruction of associates, bit such tendency is observed. Note that the absence of peak at 40 °C in melting curves for DNA–Ag–Phen complexes in 0.005 M NaNO_3_ shows that Ag–Phen molecules are associated with DNA. At *C*(Ag–Phen) < 4 × 10^−6^ M, we do not observe the hyperchromism at high temperature, which shows that the associates (stacks or dimeric structures) are formed at higher *C*(Ag–Phen). Exactly these structures are destroyed with the increase of temperature that can be observed due to the emerging of hyperchromic effect. The melting curves of DNA in complexes with Ag–Phen have no peculiarities near 40 °C. Only small gradual increase in absorption is fixed before DNA melting with the sharp increase in absorption in a rather narrow temperature region. Thus, the melting temperature of DNA in complexes was determined using first derivative of the melting curves (see Figure S2B in Supplementary Materials). We can conclude that the binding stabilizes phenanthroline associates or prevents their formation. These results indicate that binding does not lead to destabilization of DNA secondary structure. On the contrary, a slight increase in the melting temperature with increasing concentration of the compound was observed. At the same time, the width of DNA transition to the molten state increases.

**Figure 6:**
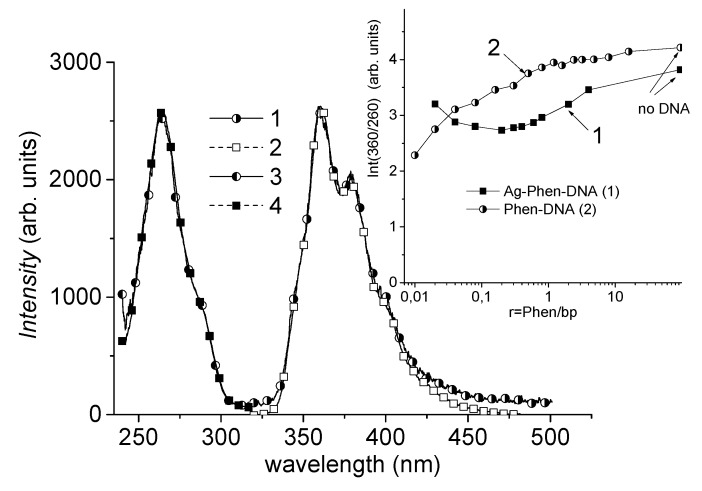
The luminescence of Ag–Phen and Phen complexes with DNA in 0.005 M NaNO_3_ solution: emission (1, 2) and excitation (3, 4) spectra of Ag–Phen–DNA (1, 3) and Phen (2, 4). Spectra are normalized to the maximum intensities: λ_ex_ = 260 nm, λ_em_ = 360 nm, *C*_AgPhen_ = 1.5 × 10^−6^ M, *C*_DNA_ = 2 × 10^−5^ M (bp), *C*_Phen_ = 3 × 10^−6^ M. The insert shows dependencies of Ag–Phen and Phen luminescence intensities at 360 nm (λ_ex_ = 260 nm) on relation of Phen to DNA base pairs concentrations (*C*_Phen_ = 3 × 10^−6^ M = const).

The luminescence of DNA solutions with Ag–Phen and Phen is presented in Figure 6, which shows an excitation spectrum of Ag–Phen–DNA solution with a peak at 265 nm and emission spectrum with the main peak at 360 nm. The shape of luminescence spectra does not change with an increase of the DNA concentration in a solution, but the intensity does depend. It is clear that the luminescence of Ag–Phen–DNA complexes is completely determined by luminescence of phenanthroline. The insert in Figure 6 shows the dependence of Ag–Phen and Phen luminescence intensities at 360 nm (λ_ex_ = 260) in DNA solution on the relation of phenantroline to DNA bp concentrations, which shows the interaction between phenanthroline and DNA. The monotonous change in luminescence of Phen intensity in DNA–Phen complexes most likely indicates one type of binding, whereas the dependence of its intensity for Ag–Phen–DNA complexes clearly indicates two types of Ag–Phen binding with DNA, More precisely, a different state of phenanthroline during the formation of two types of Ag–Phen complexes with DNA. These data confirm the conclusion about the existence of two types of DNA–Ag–Phen complexes.

**Figure 7:**
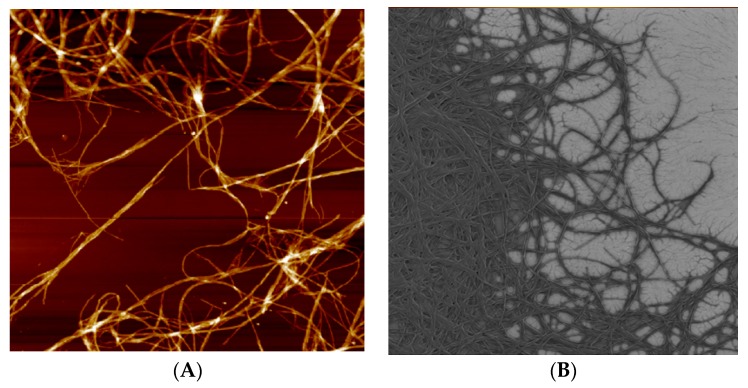
AFM (**A**); and SEM (**B**) images of DNA in complexes with Ag–Phen on silicon. Fixation was carried out from DNA solution in 0.005 M NaNO_3_ at *C*(DNA) = 0.005% and *C*(Ag–Phen) = 4.5 × 10^−4^ M; Images have a size 5 μm × 5 μm.

Let us analyze AFM and SEM images of DNA in complexes with Ag–Phen and Phen on a silicon surface (Figure 7). It was shown that after fixation of complexes on silicon surface the long filaments with parallel DNA strands were observed. The formation of filaments shows that Ag–Phen binding to DNA induces the emergence of parallel DNA chains connected to each other via common ligands. Increasing the rigidity of DNA during the formation of complexes, as well as the location of associationable phenanthroline ligands at the periphery of the helix, helps this. Elongated structures with DNA–Ag–Phen complexes can be used as a template for DNA metallization, e.g., for the usage in nanoelectronics. Indeed, the binding of Ag–Phen to DNA produces the points of silver reduction on macromolecule. 

**Figure 8:**
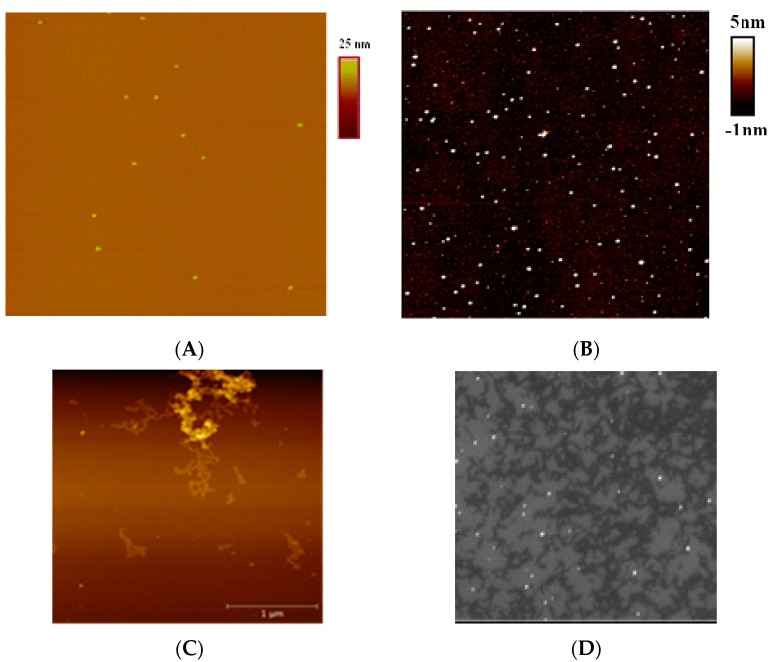
The result of silver reduction in: AgNO_3_ (**A**); and Ag–Phen (**B**–**D**) solutions. AFM (**A**–**C**); and SEM (**D**) images of nanoparticles on silicon after their fixation from the solution with *C*(Ag–Phen) = 2 × 10^−5^ M (**A**,**C**,**D**) and 8 × 10^−5^ M (**C**) at *C*(NaBH_4_) = 1.25 mM. (**A**,**C**,**D**) Images have a size 3 μm × 3 μm, (**B**) image has a size 5 μm × 5 μm.

The results of silver reduction in Ag–Phen solution without DNA and with NaBH_4_ are presented in Figure 8. AFM and SEM images show nanoparticles with the size about 30–40 nm on a silicon surface. It was shown that the reduction of silver ions in AgNO_3_ solution causes the emergence of particles with similar size (Figure 8A). At high Ag–Phen concentration the reduction of silver is followed by the emergence of associates of Ag–Phen containing reduced silver (Figure 8C).

Three forms of chemicals [Ag(Phen)_2_]^+^, [Ag(phen)(H_2_O)_2_]^+^ and [Ag(Phen)(AcO)] may be subjected to chemical reduction by NaBH_4_. Previously [58], the behavior of [Ag(Phen)_2_]OAc·H_2_O, [Ag(Phen)OAc]·H_2_O complexes was investigated with respect to the chemical reducing agent, such as NaBH_4_ not only in solutions, but also at the “natural polymer MCC-solution” interface (MCC is microcrystalline cellulose). It was found that in these systems, along with 1,10-phenanthroline derivatives of Ag(I), there are observed the stabilized nanoclusters of silver (0), while the MCC matrix promotes occurrence of such clusters.

**Figure 9:**
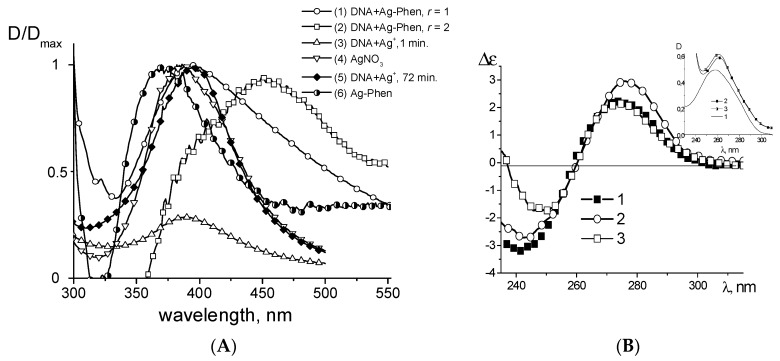
(**A**) Plasmon resonance peak (relative change) that appears as a result of silver reduction with NaBH_4_ in AgNO_3_ solution (4) after the adding of NaBH_4_ into DNA solution with AgNO_3_: in 1 min (3) and in 72 min (5); the result of silver reduction in Ag–Phen solution (6), after formation of Ag–Phen complexes with DNA at *C*(DNA) = 0.0035%, *C*(Ag–Phen) = *C* (NaBH_4_) = 1.2 × 10^−4^ M, *r* = 2 (2); and at *C*(DNA) = 0.008%; *C*(Ag–Phen) = 1.14 × 10^−4^ M; *C*(NaBH_4_) = 1.5 × 10^−3^ M, *r* = 1 (1). (**B**) CD spectra for DNA (1), DNA in complexes with Ag–Phen (2) and same solution after the reduction of silver (3); insert: UV absorption spectra for same solutions. *C*(DNA) = 0.0025%, *C*(Ag–Phen) = *C*(NaBH_4_) = 0.5 × 10^−5^ M, *r* = 0.4.

The reduction of silver after the formation of Ag–Phen complexes with DNA in a solution was carried out using sodium borohydride. The colloidal solution of silver nanoparticles with the size about 30 nm (Figure 8) has a plasmon peak at (387 ± 1) nm that appears after adding the reducer into AgNO_3_ solution without DNA (Figure 9A). Similar result was observed in DNA solution with AgNO_3_ just after the addition of sodium borohydride. However, the emergence of a plasmon peak at 387 nm (with low intensity) in a minute followed with its increase and bathochromic shift to (395 ± 1) nm in 75 min (Figure 9A, curves 2 and 5). We can assume that at the first stage the reduction of free Ag^+^ ions (not bound to DNA) occurs. With time (about 72 min) the nanoparticles are formed directly on DNA strands, and bathochromic shift of plasmon peak is observed. In this case, Ag^+^ ions bound with DNA nitrogen bases and act as the points of Ag^0^ growth. Therefore, there are two plasmon peaks, which show two types of nanoparticles in DNA solutions: nanoparticles formed on DNA (peak at 395 nm) and free nanoparticles in a solution (peak at 387 nm). Adding NaBH_4_ after the formation of Ag–Phen complexes with DNA at *r* = 1 (1 molecules per 1 base pair) causes the emergence of a plasmon peak at (394 ± 1) nm (silver reduction on DNA). At lower DNA concentration (*r* = 2) the wide plasmon band with the prevailing peak at (450 ± 1) nm and unresolved peak about 387–394 nm are observed as a result of silver reduction on DNA in the 1,10-phenanthroline environment.

It was shown that after silver reduction in a solution with DNA–Ag–Phen complexes, the CD spectrum of DNA returns to the spectrum recorded for a free DNA (Figure 9B). Segmental optical anisotropy and hence the persistent length of DNA do not change noticeably (Figure 4). Therefore, silver reduction really affects Ag–Phen complexes with DNA (especially the orientation of phenanthroline around the DNA helix). This process results also in decreased viscosity of DNA solution with Ag–Phen (Figure 4A). DNA metallization does not significantly affect DNA secondary structure (as proven by CD spectra), but viscosity shows that we must assume that DNA metallization at not high Ag–Phen concentration causes a decrease in DNA swelling. The polyelectrolyte swelling does not change significantly in the DNA solution with similar AgNO_3_ concentration (Figure 4A). Thus, we relate low viscosity of DNA–Ag–Phen solutions with volume effects due to change in polymer-solvent interactions. Indeed, high Ag–Phen concentration induces the precipitation of DNA fibrils. 

**Figure 10:**
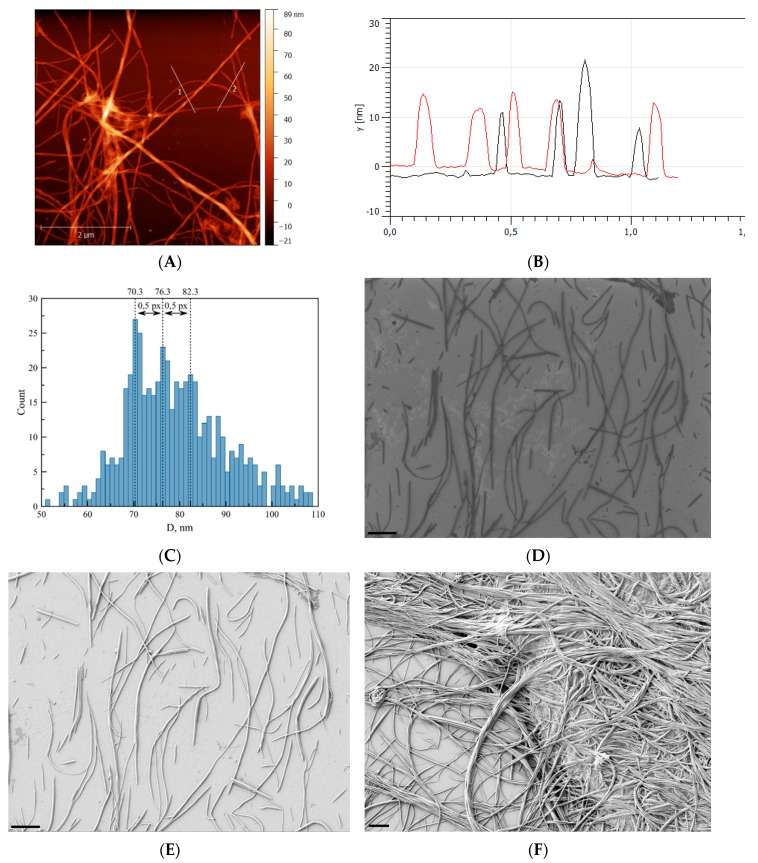
(**A**,**B**) AFM results; and (**D**–**F**) SEM images of fibrils after silver reduction in solution with DNA–Ag–Phen complexes (*C*(DNA) = 0.005%, *C*(Ag–Phen) = *C*(NaBH_4_) = 4.5 × 10^−4^ M in 0.005 M NaNO_3_). After fixation on: mica (**A**); and silicon (**D**–**F**) surfaces. Image (**D**) is obtained by InLens detector, image (**E**) by SE2. The scale bar is 1 μm. Diagram (**C**) shows the distribution of diameters of fibrils on image (**E**) (five different images).

AFM images of DNA–Ag–Phen complexes after reduction of silver demonstrate similar filaments, which was observed without silver reduction (Figure 7A and Figure 10A). Such fibrils appear in DNA solution after silver reduction at *C*(Ag–Phen) > 3 × 10^−4^ M at DNA concentration >0.006%. As a result of precipitation of Ag–Phen–DNA complexes, a white suspension forms in the solution. After a day, NaBH_4_ was added to this solution (in a solution *C*(NaBH_4_) = 4.5 × 10^−4^ M). Over time, the structures in a solution darken up to black, while the surrounding solvent remains transparent and colorless. 

It was shown by AFM, that after the reduction of silver the thickness of fibrils becomes approximately twice as large. Unfortunately, AFM images do not really give direct information about whether the recovered silver contains fibrils if the height of such metallic inclusions is not high enough relative to the difference in height of fibrils themselves. At the same time, spectroscopic data, the color of the fibrils when they are sampled from the solutions, SEM and TEM images indicate the presence of the reduced silver just on the DNA. We previously studied in detail the reduction of silver on DNA using silver nitrate [37,38]. In this case, the silver reduction contributes to the emergence of discrete silver nanoparticles on DNA strands. Homogeneous and stable DNA fibrils form easily when we use Ag–Phen. It is very tempting to get such structures covered with a thin layer of silver. In particular, this can be used to create plasmon waveguides. The presence of heterocyclic phenanthroline ligands in Ag–Phen provides a more homogeneous coating of DNA fibrils with silver without the formation of discrete nanoparticles. The plasmon peak in a solution containing such structures is about 460 nm (after silver reduction in Ag–Phen solution, plasmon peak is observed at 370 nm).

To obtain the separated structures shown in Figure 10D,E with SEM technique, precipitate was removed from the solution and washed on the surface of silicon. An analysis of fibrils’ distribution obtained over the thickness showed that structures with a diameter of about 100 nm predominate (Figure 10D,E). The resulting separated fibrils were statistically examined using the FiJi program according to the algorithm described in [59]. At high Ag–Phen concentration, we assume the formation of ordered Phen ligands from the outer side of the DNA helix. Phenanthroline ligands are inclined to formation of stacked structures in a water solution and ensure DNA precipitation after silver reduction. The binding of Ag–Phen to DNA favors DNA metallization, which is different from that observed after reducing Ag^+^ ions bound to DNA in AgNO_3_ solution, when we see nanoparticles localized on a macromolecule [37,38]. We cannot see small nanoparticles in DNA–Ag–Phen solutions after silver reduction (but spectral data show that metallization is realized on DNA). We assume that upon reduction of silver after formation of DNA complexes with Ag–Phen, the DNA fibrils (bundles) formed on the surface of hydrophobic silicon are coated with a thin layer of silver. Metallic silver can be also within the fibrils. 

On almost all large-scale images of fibrils, after the silver reduction, light areas on a dark background of organic fibrils were identified, which could be regions with a high concentration of reduced silver. However, light areas in the images can also be caused by edge and/or charge effects when conducting electron microscopy measurements. Evaluating the results as preliminary, we would like to demonstrate that the formation of fibrils can be used for further metallization of DNA, which is undoubtedly evidenced by spectral data. Experimental results show the emergence of more stable and thicker fibrils after silver reduction using DNA–Ag–Phen complexes, as well as the possibility of DNA metallization in such systems. Indeed, preliminary studies using TEM technique shows that the reduced silver is present in the fibrils. In addition, the partial reduction of silver in complexes occurs without the addition of a reducing agent (see Figure S3 in Supplementary Materials).

Our research has shown that the use of certain concentrations of the Ag–Phen compound in a DNA solution provides the formation of stable fibrils that can be used, for example, for various applications in nanotechnology development. Silver provides coordination of the compound to DNA, and phenanthroline promotes the formation of interchain contacts for the formation of fibrils. An increase in the persistent length of DNA upon binding also assists the orientation of the double helices in fibrils. The advantage of DNA metallization in such structures is as follows. Firstly, the structure of fibrils and the fixation of silver on DNA provides regularly located growth points for silver reduction. Secondly, phenanthroline helps stabilize the reduced silver. Indeed, when the silver is reduced in Ag–Phen solutions, small uniform particles are formed, for which a narrow peak of the plasmon resonance is observed. Silver nanoparticles are not visible on DNA–Ag–Phen fibrils. Nevertheless, when silver nitrate is added to the solution after the formation of DNA fibrils, we can see silver bunches on fibrils (see Figure S4 in Supplementary Materials). This indicates that there are no enough vacant binding sites for silver ions on DNA in this case. 

Spectral data demonstrate that metallic silver is located on DNA fibrils. It can be localized either inside the fibrils, or as a thin layer on the surface. Certainly, DNA metallization in complexes with Ag–Phen should be done in details.

We should point out that in silver concentration less than the concentration of G + C pairs of DNA, the so-called nanoclusters consisting of a small amount of atoms (approximately up to 20) are formed instead of nanoparticles on a DNA molecule as a result of silver reduction [31]. Clusters do not show a peak of plasmonic resonance, but they are capable of luminescing upon excitation in UV spectrum range. We checked the possibility of silver cluster formation on DNA–Ag–Phen structures. In our experiments, we did not observe such clusters (the luminescence of clusters only slightly overlaps the spectrum of phenanthroline).

## 4. Conclusions

Using a set of experimental methods that gave knowledge about the binding of Ag–Phen to DNA, we analyzed the influence of complexes on the secondary and tertiary structure of the macromolecule. The information on the spectral properties of DNA and Ag–Phen in the complexes; the stability of DNA secondary structure during the interaction; the size, optical anisotropy and rigidity of DNA molecule during the binding; and the shape, size and other characteristics of the structures were obtained. Spectral data show that there exist two types of Ag–Phen–DNA complexes in solutions. First type is observed at low Ag–Phen concentration in DNA solution. The coordination of silver atoms to DNA releases Phen ligands that interact with DNA as intercalators. The second type of Ag–Phen binding to DNA appears at high Ag–Phen concentration with the formation of phenanthroline associates on DNA outside base pairs. CD spectra indicate an ordered arrangement of ligands. We can point out that the interaction of DNA with Phen ligands is present in all systems. The coordination of silver to bases of DNA is also observed. The melting of DNA in complexes with Ag–Phen shows that the binding causes a certain stabilization of DNA secondary structure. 

It was shown in our research that silver is reduced on DNA after formation of Ag–Phen–DNA complexes, alongside the formation of the structures differing from the case of silver reduction in the complexes with Ag^+^. Therefore, the Ag–Phen compound is interesting not only as a biologically active substance, but also as an agent facilitating DNA metallization. Further experiments would allow selecting the optimal ways for the formation of nanostructures based on DNA metallization in different manners.

## Figures and Tables

**Table 1:** Melting temperature *T*_m_ of DNA in complexes with Ag–Phen, Phen and Ag^+^ in 0.005 M NaNO_3_.

Concentration of Compound in DNA Solution	DNA–Ag–Phen	DNA–Ag^+^	DNA–Phen
0	65 ± 1	65 ± 1	65 ± 1
1.5 × 10^−6^ M	67 ± 2	66 ± 1	64 ± 1
3 × 10^−6^ M	68 ± 2	68 ± 2	64 ± 1
6 × 10^−6^ M	74 ± 4	73 ± 3	63 ± 2

## Supplementary Materials

Supplementary Materials are available online at www.mdpi.com/2073-4360/9/6/211/s1.
