# Supplementary material for: DNA Binding with Acetate Bis(1,10-phenanthroline)silver(I) Monohydrate in a Solution and Metallization of Formed Structures
Source: Polymers (Basel). 2017 Jun 8;9(6):211. doi: 10.3390/polym9060211 (PMC6432125; doi:10.3390/polym9060211)
Supplement: Supplementary file 1 [file polymers-09-00211-s001.pdf]

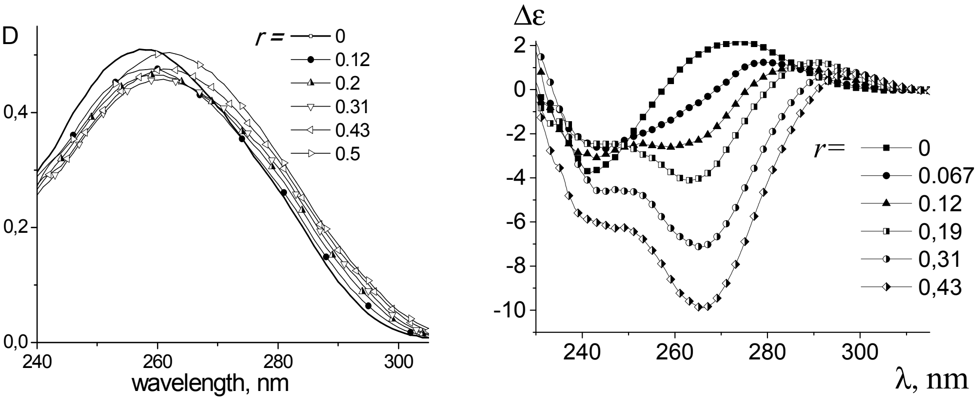

A B

Figure S1. Normalized absorption spectra (A) and CD spectra (B) of DNA in AgNO3 solutions. The values of *r* are shown near lines.

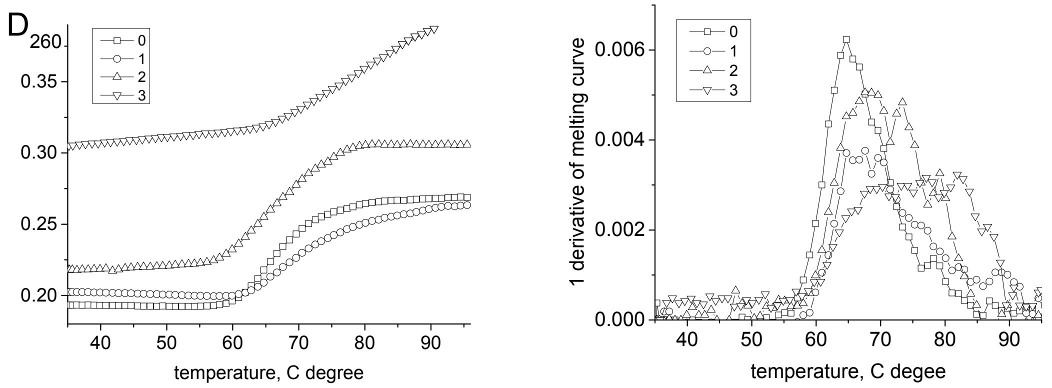

A B

Figure S2. Melting curves (A) and their first derivatives (B) for DNA-Ag-Phen complexes at C(Ag-Phen)=0 (0), 1.5×10^{-6} M (1), 3×10^{-6} M (2), 6×10^{-6} M (3).

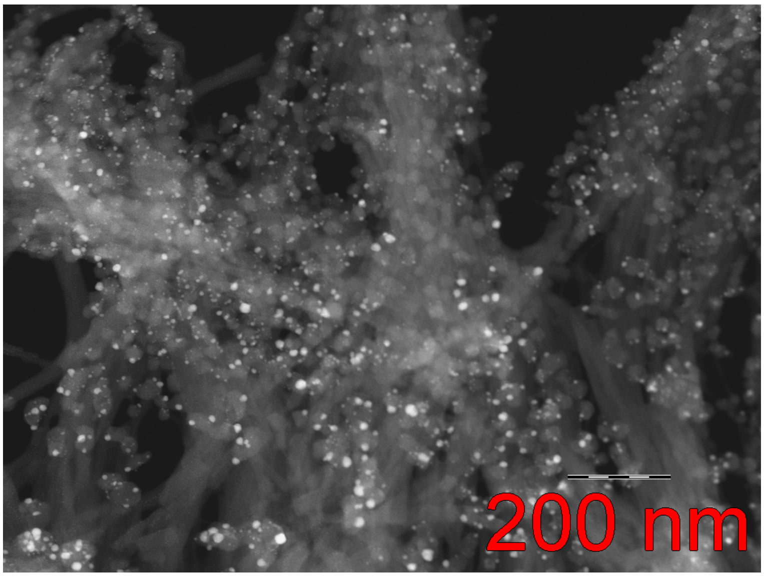

Figure S3. TEM image of fibrils after silver reduction. EDX measurements showed that the light particles consist of silver. As can be seen, regions saturated with silver are formed, which differ from dense particles. We can assume that either loose clusters inside fibrils or a film on their surface are presented .

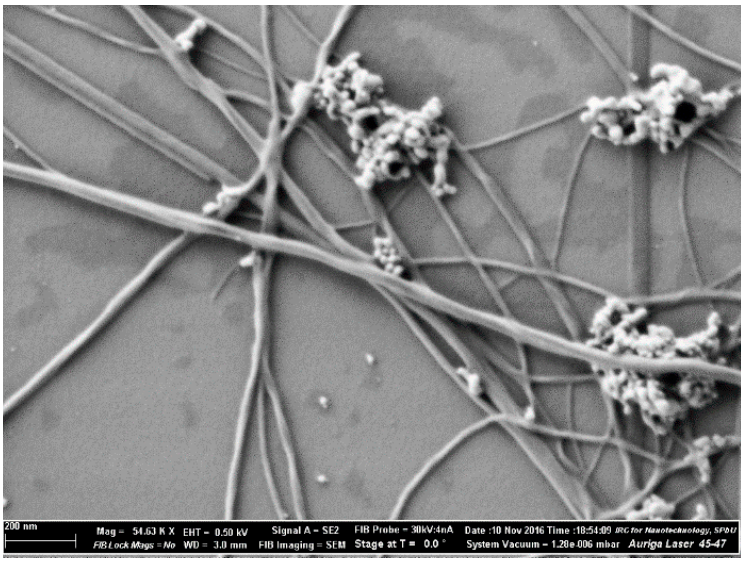

Figure S4. The result of silver reduction on DNA fibrils with an addition of $AgNO_3$ to solution with fibrils at C(AgNO3)=C(Ag-Phen)= $4{,}5 \cdot 10^{-4}$ M in DNA solution.
